# Effectiveness of an Online Peer Gatekeeper Training Program for Postsecondary Students on Suicide Prevention in Japan: Protocol for a Randomized Controlled Trial

**DOI:** 10.2196/34832

**Published:** 2022-04-26

**Authors:** Kyosuke Nozawa, Ayaka Ishii, Hiroki Asaoka, Mai Iwanaga, Yousuke Kumakura, Yuri Oyabu, Tomohiro Shinozaki, Kotaro Imamura, Norito Kawakami, Yuki Miyamoto

**Affiliations:** 1 Department of Psychiatric Nursing Graduate School of Medicine The University of Tokyo Tokyo Japan; 2 Specified Nonprofit Corporation Light Ring Tokyo Japan; 3 Department of Mental Health Graduate School of Medicine The University of Tokyo Tokyo Japan; 4 Department of Information and Computer Technology Tokyo University of Science Tokyo Japan

**Keywords:** gatekeeper, suicide prevention, mental health, youth, school, student, peer support, depression, self-efficacy, suicide, prevention, online training, online program, protocol, RCT, control trial

## Abstract

**Background:**

Postsecondary student suicide is one of Japan’s most severe public health problems. Gatekeeper training (GKT) programs are a generally recommended suicide prevention intervention in Japan. For suicide countermeasures, an online program tailored to students may enhance self-efficacy as a gatekeeper.

**Objective:**

This study aims to describe a research protocol to investigate the effect of a newly developed internet-delivered online peer GKT program to improve postsecondary student self-efficacy as gatekeepers for suicide countermeasures in Japan.

**Methods:**

This study is a 2-arm, parallel, randomized controlled trial with a 1:1 (intervention: waiting list) allocation. Participants (n=320) will be recruited, and those who meet the inclusion criteria will be randomly allocated to the intervention or waiting list control group. An approximately 85-minute, 6-section, internet-based gatekeeper program for postsecondary students has been developed that includes videos to help participants acquire skills as gatekeepers. The intervention group will complete the program within 10 days. The primary outcome, self-efficacy as a gatekeeper, is measured using the Gatekeeper Self-Efficacy Scale at baseline, immediately after taking the program, and 2 months after the survey after completing the program follow-up. To compare the primary outcomes, a *t* test, where the significance level is 5% (2-sided), will be used to test the intervention effect on an intention-to-treat basis.

**Results:**

The study was at the stage of data collection at the time of submission. We recruited participants for this study during August and September 2021, and data collection will continue until December 2021. The data analysis related to the primary outcome will start in December 2021, and we hope to publish the results in 2022 or 2023.

**Conclusions:**

This is the first study to investigate the effectiveness of an online GKT program for postsecondary students to improve self-efficacy as a gatekeeper using a randomized controlled trial design. The study will explore the potential of an online peer gatekeeper program for postsecondary students that can be disseminated online to a large number of students with minimal cost.

**Trial Registration:**

University Hospital Medical Information Network Clinical Trials Registry UMIN000045325; https://upload.umin.ac.jp/cgi-open-bin/ctr/ctr_view.cgi?recptno=R000051685

**International Registered Report Identifier (IRRID):**

DERR1-10.2196/34832

## Introduction

### Background

According to the World Health Organization, every year about 700,000 people die by suicide [1]. Suicide is one of the most severe public health problems in the world. The high number of suicides in Japan has been a national issue since it transcended 30,000 per year in 1998 [2]. The number of suicides in 2020 was 21,081, an increase of about 4.5% compared to 2019 [3]. Although the number of suicides and suicide rate are declining compared to historic peak times in Japan, the Japanese suicide rate is the 5th highest among Organisation for Economic Co-operation and Development countries; additionally, the Japanese suicide rate is the highest among all Group of 7 (G7) members [4].

Furthermore, suicide was the leading cause of death among individuals aged 15 to 39 years in Japan [3]. This situation in Japan is serious, and among all G7 members, it is only in Japan that the leading cause of death among youth aged 15 to 34 years is suicide [5]. Suicide is the leading cause of death for college and university students in Japan [6]. Therefore, suicide prevention measures for young people, especially students, are urgently needed.

Recently, COVID‐19 has spread throughout the world. Problems associated with outbreaks of infections such as COVID-19 have an undesirable impact on learning and can also lead to mental health problems [7]. The effects of COVID-19 on the already-high suicide rate create an urgent need to deal with suicide among youth, especially students. A previous study showed that about 80% of college students who died by suicide had no prior contact with campus mental health professionals, and 85% of college students with moderate to severe depression did not get treatment [6,8]. Considering this fact, it is possible that students who have mental health problems cannot take measures by themselves (ie, help-seeking) or are not aware of the severity of their own condition. Therefore, awareness by other persons, such as gatekeepers who can detect and refer at-risk individuals, is essential for students experiencing difficulties.

A gatekeeper is someone aware of the signs of suicide who can take appropriate action (ie, be aware of people in need, speak to them, listen closely to them, direct those in need to resources for support, and watch over them) [9]. Gatekeeper training (GKT) is a program to foster the development of gatekeepers and aims to help nonprofessionals identify and respond to those at risk of suicidal behavior; it is the most general intervention in suicide prevention [10]. GKT programs are a generally recommended suicide prevention intervention in Japan, and the Japanese government and all local governments in Japan presently conduct GKT in various forms [11]. GKT builds the self-efficacy of those acting as a gatekeeper, such as speaking to people at risk of suicide. A recent meta-analysis in the United States reported the effectiveness of GKT in universities on suicide prevention knowledge, skills, and self-efficacy [12]. Thus, increasing a student’s self-efficacy as a gatekeeper leads to more gatekeeper actions and, as a result, suicide prevention.

Students, however, may encounter obstacles in attending a GKT program. The most significant hurdle for students may be a lack of time and resources to participate in face-to-face training sessions. In addition, GKT programs conducted at a school or in a class unit often involve familiar acquaintances, and as GKT also includes private content such as sharing experiences and opinions in group work, some may not want to participate due to privacy concerns. As such, on-demand online GKT programs may be helpful in overcoming such hurdles for students. On-demand online GKT provides easy, fast, instant, and privileged access to needed knowledge about student suicide prevention at any time and from any location.

Most university GKT programs focus on teachers and related staff (eg, school counselors, nursing teachers) because of their daily interaction with students [13]. However, students have more opportunities to interact with other students than faculty members. Students may feel it is more challenging to talk to faculty staff than to other students (acquaintances). Suicidal college students tend to seek help from friends and family rather than from a mental health professional [14]. Past studies have shown that college students are most likely to talk to friends about suicidal ideation or ask for treatment when recommended by a friend [15,16]. Hence, students are especially advantageous as gatekeepers. GKT tailored to students is needed to increase the number of student gatekeepers.

Few programs have reported on the effectiveness of GKT programs for decreasing the number of suicides. Most studies have reported on suicide knowledge, attitudes, and skills of trainees instead of the number of suicides [17,18]. It is difficult to evaluate the effectiveness of GKT programs based on a decrease in suicide attempts or suicides. Therefore, in this study, we decided to use a surrogate outcome.

Behavior as gatekeeper has been conceptualized with the theory of planned behavior (TPB) [19,20]. According to TPB, “attitudes toward the behavior” may be our thoughts about the potency of, and our emotional reaction against, conducting the behavior [21]. TPB shows “perceived behavioral control” is the perceived controllability of a specific behavior, including a person’s understanding and ability to perform the behavior [21]. These affect “intention to intervene” and lead to actual actions [21].

We hypothesized that self-efficacy as a gatekeeper affects attitudes toward the behavior and perceived behavioral control from this theory, leading to actual behavior as a gatekeeper. Past research has reported that GKT programs have improved self-efficacy as a gatekeeper [22-25]. Furthermore, self-efficacy was chosen as the primary outcome in this study because we thought self-efficacy in attitudes has the most significant impact on behavior among the commonly used surrogate items (knowledge, attitudes, and skills).

Assuming that encouraging action for suicide countermeasures as a gatekeeper lowers the suicide rate, a GKT program was created aimed at increasing self-efficacy, which affects behavior as a gatekeeper based on the attitude of TPB.

### Objectives

To date, to the best of our knowledge, there has been no online GKT program for students in Japan. Moreover, no randomized controlled trials (RCTs) have been conducted to verify the effectiveness of online GKT programs for students. Accordingly, the aims of this research are to (1) create an online peer GKT program for students and (2) verify its effectiveness through RCTs.

## Methods

### Trial Design

**Figure 1:**
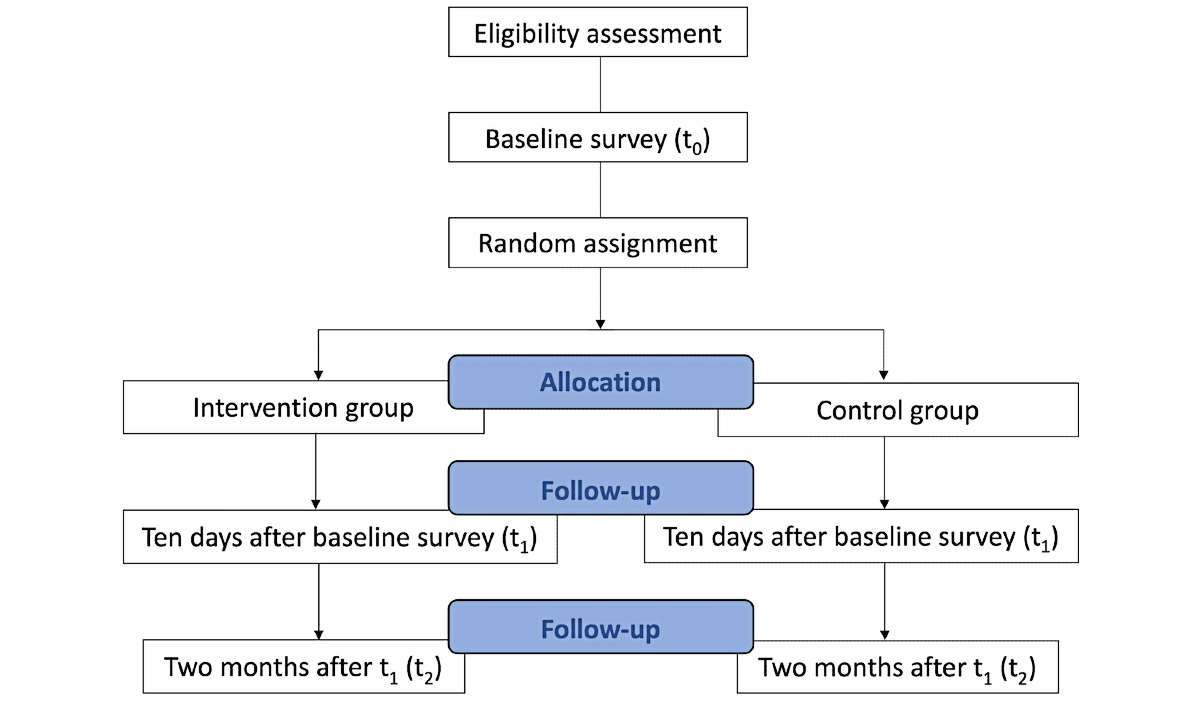
Participant flowchart.

Figure 1 shows the participant flow and study design. This study will be a 2-arm parallel-group nonblinded RCT with a GKT intervention group and a waitlist control group. Eligible participants are required to complete the baseline survey (*t*_0_) and are randomly allocated at a 1:1 ratio either to the intervention group or the control group. All surveys will be conducted via online questionnaires. Online follow-up surveys will be implemented about 10 days after the *t*_0_ survey (*t*_1_: immediately after the intervention), and at 2 months after the *t*_1_ survey (*t*_2_). After the 2-month follow-up, the GKT program will be provided to participants in the control group who wish to attend. This manuscript has been written according to the Standard Protocol Items: Recommendations for Interventional Trials (SPIRIT) guidelines [26].

### Participants and Setting

This study will target postsecondary school students (eg, college and university students) in Japan. The inclusion criteria for individuals will be students aged 18 to 29 years, in Japanese postsecondary school, with access to the internet via their own PC, smartphone, or tablet, and who acknowledge the program offered is a suicide prevention program. The exclusion criteria will be students who cannot read and write Japanese or who are full-time workers. The researchers asked their acquaintances to publicize this study through the mailing lists of their institutions. We also used social networking sites, our websites, and flyers to inform this study.

### Procedure

Candidates will click the URL provided in the information about the invitation for GKT research and read a full explanation of the purpose and procedures of the study on the website. Students who are interested in participating will be asked to mark the consent option and type their name and their email address to give their consent. This consent information will be sent to the research center and preserved. Students who are not interested in participating will be asked to leave the website. Subsequently, participants who submitted the consent information will complete the baseline survey. After completing the baseline questionnaire, participants will be randomly assigned either to the intervention or the control group. Researchers will inform participants of their group assignment and send an email containing the GKT program’s homepage URL and password to participants in the intervention group. Participants in the intervention group will click the URL, enter the password, and receive the GKT program. They will be asked not to share this URL, password, and the GKT program content with others. Participants in the intervention group will be instructed to take the program within 10 days of receiving notification of group assignment. *t*_1_ surveys will be administered to both the intervention and control groups. Participants in the intervention group are instructed to complete the *t*_1_ survey immediately after attending the program. Participants in the control group are asked to respond to the *t*_1_ survey 10 days after the *t*_0_ survey. Those in the control group will receive the GKT program after the *t*_2_ survey. Participants who answer all 3 surveys will get the chance to win a ¥1200 (US $10.14) gift card from Amazon as a monetary incentive to promote retention and follow-up completion by lottery.

### Intervention Program

**Figure 2:**
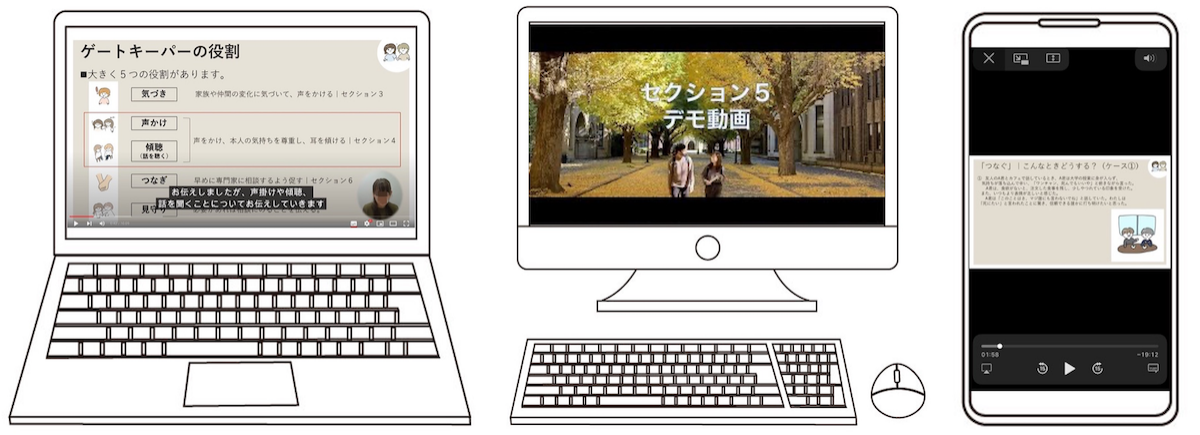
Screenshots of the internet-based gatekeeper training program designed for postsecondary students.

We developed a new GKT program using the following steps. First, we decided upon the composition of the program content by referring to existing GKT programs and the role of the gatekeeper advocated by the Ministry of Health, Labor, and Welfare. The first author then developed a preliminary program in consultation with researchers specializing in psychiatric nursing and psychiatry and in collaboration with experts in suicide prevention from a Japanese nonprofit organization. Next, the first author conducted a feedback session with 5 students and modified the program according to their recommendations. Last, a pilot trial was performed with vocational school students nested in one organization (n=18). Additionally, an opinion exchange meeting was held at the workshop for people involved in mental health and welfare to watch a part of the GKT and give their impressions. Based on these opinions and the results of the pilot trial, we modified the program and created the final version of the GKT program. The final version of the internet-based GKT program designed for postsecondary students was developed (Figure 2). The home page of the GKT program is the platform for participants to access the program. The content of this homepage includes an explanation of how to use the GKT program, videos of the GKT program (sections 1-6) via YouTube, a comment section (online discussion board), a text download page, and a contact form to the administrator. The videos of the GKT program consist of the following 6 sections for a total of approximately 85 minutes (Table 1). The GKT program covers mental health basics (section 1), current status of suicide problems (section 2), danger sign features of suicide (section 3), how to appropriately respond (section 4), demo video (section 5), and referral information for appropriate resources (section 6). Each section takes 10-20 minutes to view and contains a voiceover, cases, personal work, and quizzes (except for section 5). We made videos of the GKT program for uploading to YouTube. Sections 1-4 and 6 were created by us in PowerPoint with lecture-style videos. Section 5 was shot with a video camera and edited with video editing software. The GKT program introduced a demo video (section 5) instead of role-playing. The demo video shows how to respond in situations students are likely to experience, and participants can learn how to respond concretely by imagining the characters appearing in the examples and replacing them with themselves. These are online videos but are unlisted and restricted to people who have the link to the video so only participants in the intervention group and researchers can view them. Because they are played on YouTube, the modules were designed so the participant controlled every aspect of the module, such as skipping or replaying a movie, adjusting playback speed, and switching the voiceover or subtitles on or off. Of course, during the intervention period, participants could watch the video at any time and place. Participants are instructed to watch sections 1 to 6 in order. Furthermore, participants have access to an online discussion board on the website, which gives them the opportunity to exchange thoughts with each other. On this online discussion board, participants can browse other participants’ ideas and write their own opinions, but cannot reply to other participants’ comments.

This GKT program does not have homework and is fully self-guided. However, participants are encouraged to voluntarily comment on their personal work responses and section impressions and read the opinions of other participants. Participants can download the text used in the videos of the GKT program on this homepage. In each section, an announcement is made to stop watching the video if they feel uncomfortable. The pages in each section provide a list of contacts available if they feel unwell.

**Table 1:** Contents of the online peer gatekeeper training program.

Section	Theme	Video viewing time	Contents
1	Mental health basics	09:59	Concepts of the gatekeeper Psychology of people who are mentally ill Basic knowledge about depression
2	Current states of the suicide problem	12:56	Statistics of suicide Social isolation The importance of consulting
3	Recognizing suicide risk	09:54	Warning signs of suicide Risk factors for suicide
4	Questioning	10:10	How to call out How to listening
5	Role play (demo video)	19:52	—^a^
6	Referral to care	21:11	Concepts of referral to care Case study Resources available for suicide countermeasures

^a^Not applicable.

### Outcomes

Table 2 summarizes the outcome measures. Only the participants in the intervention group are assessed regarding the process evaluation outcomes (eg, usability, satisfaction) at the *t*_1_ survey. All data are collected using web-based self-report questionnaires. At the *t*_1_ and *t*_2_ surveys, the researcher (KN) will send at least 2 emails reminding nonrespondents to complete the questionnaires.

**Table 2:** Assessment schedule of the outcome measures for the randomized controlled trial for the students’ online peer gatekeeper training program.

Measurement			Aim		*t*_1_		*t*_2_		*t*_3_
**Primary outcome**									
	GKSES^a^	Self-efficacy as gatekeeper		✓		✓		✓	
**Secondary outcomes**									
	Short form of the LOSS^b^	Literacy of suicide		✓		✓		✓	
	SOC3-UTHS^c^	Sense of coherence		✓		✓		✓	
	MIDUS^d^	Stigma		✓		✓		✓	
	Scale for measuring help-seeking styles	Help-seeking		✓		✓		✓	
	K6^e^	Psychological distress		✓		✓		✓	
	Rosenberg Self-Esteem Scale–Japanese version	Self-esteem		✓		✓		✓	
	Tachikawa Resilience Scale	Resilience		✓		✓		✓	
	ACT	Behavior as gatekeepers		—^f^				✓	
**Process evaluation**									
	iOSDMH^g^	Acceptability, appropriateness, feasibility, satisfaction, adverse effect				✓			
**Subgroup analysis**									
	FCV-19S^h^	Level of fear of COVID-19		✓					
	CIUS^i^	Tendency toward internet dependence		✓					

^a^GKSES: Gatekeeper Self-Efficacy Scale.

^b^LOSS: Literacy of Suicide Scale.

^c^SOC3-UTHS: University of Tokyo Health Sociology version of the Sense of Coherence–3 scale.

^d^MIDUS: Mental Illness and Disorder Understanding Scale.

^e^K6: Kessler Psychological Distress Scale.

^f^Not applicable.

^g^iOSDMH: Implementation Outcome Scales for Digital Mental Health.

^h^FCV-19S: Fear of COVID-19 Scale.

^i^CIUS: Compulsive Internet Use Scale.

#### Primary Outcome

The primary outcome is confidence in students’ gatekeeper skills, assessed using the Gatekeeper Self-Efficacy Scale (GKSES) [27]. The GKSES was developed to measure confidence in a person’s own gatekeeper skills and consists of 9 items measured on a 7-point Likert scale. The GKSES is a 1-factor structure model. All items ask about confidence in their own gatekeeper skills. An example is, “I can understand the mental states of people who intend to die by suicide.” Higher average scores for total items indicate a higher confidence in one’s own gatekeeper skill. Reliability and validity have been confirmed among Japanese. Reliability of GKSES is very good with Cronbach *α*=.95 [27].

#### Secondary Outcomes

##### Literacy of Suicide Scale

The Japanese version of short forms of the Literacy of Suicide Scale (LOSS) will be used to assess suicide literacy; a 12-item short form of LOSS [28] will be used in this study. We obtained a license for the use of the LOSS by its creator, who also gave us a Japanese translation of the short form. We have revised the Japanese version so that students can answer it more clearly. The scale provides a total literacy score (percentage correct) and can be broken down into the 4 literacy themes of risk factors, signs/symptoms, cause/nature, and treatment/prevention. Correct responses are scored 1, while incorrect or I don’t know responses are scored 0. Literacy scores are the sum of correct items, with higher scores indicating higher suicide literacy.

##### Sense of Coherence

A sense of coherence (SOC) will be assessed using the University of Tokyo Health Sociology version of the SOC3 scale (SOC3-UTHS) [29]. SOC is defined as “individuals’ perceptions of life and resources to help them overcome hardships in life” that relate to stress management ability [29]. The scale consists of 3 dimensions: manageability, meaningfulness, and comprehensibility. All items are rated on a 7-point Likert-type scale ranging from 1 (never) to 7 (always). The Japanese version of the SOC scale’s reliability and validity was verified in a previous study. Cronbach alpha for SOC3-UTHS ranges from .80 to .98, indicating acceptable to high internal consistency [29]. The score is the average of the 3 items, which is then used for analyses. A higher score indicates a higher SOC.

##### Stigma

To assess community understanding of mental health and the stigma associated with mental illness and disorder, we will use the Mental Illness and Disorder Understanding Scale (MIDUS), consisting of 15 items measured on a 5-point Likert scale [30]. MIDUS has 3 factors: treatability of illness, efficacy of medication, and social recognition of illness. Lower total scores indicate better understanding. MIDUS’s reliability and validity were verified in a previous study. Reliability of MIDUS is very good with Cronbach *α*=.78 [30].

##### Help-Seeking

To measure help-seeking, we will use a scale for measuring help-seeking styles [31]. The degree of help-seeking behaviors will be assessed by the Scale for Measuring Help-Seeking Styles, which consists of 15 items measured on a 7-point Likert scale. The scale consists of 3 dimensions: self-directed help-seeking, excessive help-seeking, and avoidant help-seeking. The Scale for Measuring Help-Seeking Styles’ reliability and validity was verified in a previous study [31]. A high total score for each factor indicates a high possibility of taking help-seeking behaviors. In this study, we used the dimensions of self-directed help-seeking consisting of 4 items. Reliability of the dimensions of self-directed help-seeking is very good with Cronbach *α*=.79 [31]. A total score of this dimension (4 to 28) was calculated and used for analyses.

##### Psychological Distress

Psychological distress will be measured with the Japanese version of the Kessler Psychological Distress Scale (K6), which asks respondents how frequently they experienced symptoms of psychological distress during the previous 30 days using 6 items (*α*=.85) [32]. Responses are rated on a 5-point Likert scale ranging from 0 (none of the time) to 4 (all of the time). A total score of these items (0 to 24) will be calculated and used for analyses.

##### Self-Esteem

Self-esteem will be measured with the Japanese version of the Rosenberg Self-esteem Scale, which consists of 10 items measured on a 4-point Likert scale. This scale is a 2-factor structure. The reliability and validity of the Japanese version of the Rosenberg Self-esteem Scale were verified in a previous study. Reliability of the Rosenberg Self-esteem Scale is very good with Cronbach *α*=.89 [33].

##### Resilience

We will use the Tachikawa Resilience Scale [34], which consists of 10 items measured on a 7-point Likert scale. This scale measures an individual’s resilience, or reactions to stressful life events. Higher scores reflect higher resilience. Reliability of the Tachikawa Resilience Scale is very good with Cronbach *α*=.82 [34].

##### ACT as Gatekeeper

We asked participants 2 months after the *t*_1_ survey about their behavior as gatekeepers for the last 2 months (see Multimedia Appendix 1 for questionnaire). We asked the frequency of the following: asking acquaintances (friends) about suicidal thoughts, distress, or depressed mood; listening to an acquaintance (friends); providing appropriate information; taking acquaintance (friends) to the right resource; and noticing suicide danger signs.

##### Demographic Characteristics

Demographic data such as sex, age, grade, school type, school location, living status, international student status, part-time job situation, whether one goes to school, online class status, state of emergency or preemergency measures for COVID-19, and experience in contact with people who have suicidal ideation will also be collected.

#### Process Evaluation

Since the number of sections each participant follows could influence the scores on the questionnaires, participants in the intervention group are asked to answer for the section they watched in the questionnaire survey after the intervention program.

For process evaluation, we use the Implementation Outcome Scales for Digital Mental Health (iOSDMH) [35]. We will use the 19 items of implementation outcomes for digital health interventions. This measurement is based on 3 important concepts: acceptability (*α*=.67), appropriateness (*α*=.78), and feasibility (*α*=.83). Adverse effects (ie, harms [*α*=.78]) of eHealth interventions, such as physical symptoms (eg, tired eyes, stiff shoulders), mental symptoms (eg, insomnia), and dangerous experiences (eg, bumping into people while looking at a smartphone), will be covered in 5 items.

#### Subgroup Analysis

##### Impact of COVID-19

The researchers collected data about fears of COVID-19 to capture the impact of COVID-19 on daily life using the Japanese version of the Fear of COVID-19 Scale (FCV-19S), as the target population was recruited during the pandemic. The scale, which consists of 7 items measured on a 5-point Likert scale, consists of 2 dimensions: emotional fear reactions and symptomatic expressions of fear. The higher the score, the stronger the fear of COVID-19. The validity of the Japanese-language version of FCV-19S was verified in a previous study. Reliability of FCV-19S is very good with Cronbach *α*=.87 [36].

##### Internet Use

To assess levels of internet addiction, we will use the Japanese version of the Compulsive Internet Use Scale (CIUS), which consists of 14 items measured on a 5-point Likert scale [37]. CIUS has 3 factors: excessive absorption, difficulty in setting priorities, and mood regulation. The higher the total score, the stronger the dependence on internet use. Reliability and validity of the CIUS were verified in a previous study. Reliability of CIUS is very good with Cronbach *α*=.93 [37].

### Sample Size Calculation

The sample size calculation is based on the difference between means of change in the primary outcome (self-efficacy as the gatekeeper) from baseline (preintervention) to postintervention (*t*_1_ survey) in both arms (complete case analyses). Based on previous studies on GKT, mean effect sizes of around *d*=0.5 are expected in per-protocol analyses for improving self-efficacy [12,22-25]. In order to detect such intervention effects in 2-tailed significance testing (*α*=.05) with a power of 80%, a sample size of 64 per group (total 128) participants is required. We assume the study dropout rate will be higher in web-based intervention and assessments compared to face-to-face research. The expected study dropout was about 60% (dropouts occur at random within each group), and this meant that 320 participants (160 per group) must be included in the study.

### Randomization and Blinding

After baseline assessment, participants will be randomly assigned to either group using the permuted block method with a random block size of 2, and they will be informed of their assigned group by the first author (KN). Randomization will be stratified by sex. The computer-generated allocation list was made by an independent researcher (YM). The enrollment is conducted by the first author (KN), and the intervention starts immediately. The means for blinding in this study are limited. An independent researcher (YM) who will not analyze data will download data from the GKT database. An independent research staff person will mask the group variable before analysis, and then researchers (KN) will analyze data that is blinded to the group variable.

### Statistical Analysis

The primary analysis for the GKSES score will be on a modified intention-to-treat principle, in which all available measurements are compared according to assigned groups. Baseline variables will be summarized by the groups using frequencies and proportions (for categorical variables) or mean and standard deviation (for continuous variables). The primary end points (difference of GKSES total score between presurvey and postsurvey) between the intervention group and the control group will be compared based on a *t* test. We will set the 2-sided significance level at 5%, and the 95% confidence interval of the average difference in differences of the scores will be estimated. Secondary end points will be compared similarly. As subgroup analysis for primary and secondary end points, we will stratify by highly dependent and lesser dependent groups for internet use, a group with high fear of COVID-19 and a group with low fear of COVID-19. To help interpretation, Cohen *d* and its 95% confidence intervals will be calculated at each assessment point; the values of 0.2, 0.5, and 0.8 are considered as small, medium, and large effects, respectively [38]. Statistical analysis will be performed using SPSS (version 27, IBM Corp) statistical software.

### Data Monitoring

This study does not have an external data monitoring committee. The first author (KN) will manage participants’ progress and completion of the intervention and the follow-up assessments.

### Research Ethics and Approval

The Ethics Committee of the Faculty of Medicine and Graduate School of Medicine of the University of Tokyo approved this study (2020234NI). Before the baseline survey, candidates will be fully informed that their participation is totally voluntary and they can withdraw consent if they want (they can send a withdrawal email to researchers). Informed consent by an explanation in the document will be conducted, and the marked consent option and full name will be obtained from all participants. Even if participants withdraw consent, they will not receive any disadvantage. In addition, they will be informed that the findings of this study will be disseminated without participants’ personal information via publication and website. The participants will be told that the web-based program does not provide emergency support on the website and will be provided with corresponding contact information that can be used in case of emergency during the program. All data collected in this study are securely stored without the participants’ personal information. Access to the data is encrypted and limited to research staff named on the ethics protocol. This study protocol was registered with the University Hospital Medical Information Network Clinical Trial Registry [UMIN000045325]. If there are important modifications of the protocol, we will obtain approval for the modifications from the Ethics Committee of the Faculty of Medicine and Graduate School of Medicine of the University of Tokyo and will revise the protocol on the trial registry website.

### Data Confidentiality

Collected data will be stored as linkable anonymized data. The principal investigator will retain access to the final dataset after the trial and assume responsibility for data integrity and accuracy of the analysis.

### Patient and Public Involvement

The first author conducted informal discussions with 5 students and nonprofit organization representatives that implement youth suicide countermeasures. Based on these conversations, adding 2 researchers to those mentioned, we modified the contents to reflect a real situation. One patient and public involvement (PPI) partner, a college student, reviewed the program’s draft and noted some points that might be difficult for general students to understand. These PPI partners will participate in a discussion of the study results and in making the implementation strategy after finishing the RCT. We described the PPI process based on the PPI handbook and reporting checklists.

## Results

At the time this paper was submitted, the study was at the stage of data collection. We recruited participants for this study during August and September 2021, and data collection will continue until December 2021. Data analysis will begin in December 2021 for the outcome variables. We expect to publish the results in 2022 or 2023.

## Discussion

### Strengths of the Study

We hypothesized that the online GKT program for students will increase self-efficacy as a gatekeeper. Moreover, we hypothesized that self-efficacy as a gatekeeper affects attitudes toward the behavior and perceived behavioral control from TPB, leading to actual behavior as a gatekeeper.

The effects of an online gatekeeper program on self-efficacy will be examined for gatekeepers among postsecondary students in Japan. To the best of our knowledge, this study is the first RCT to examine the effectiveness of online GKT programs without face-to-face sessions targeting students rather than staff or faculty for their self-efficacy as gatekeepers. This online program will allow individuals to participate without worrying about their privacy anytime, from anywhere.

### Dissemination of the Findings

An online GKT program requires less involvement by mental health specialists such as public health nurses. It can be provided at a low cost in a low-resource setting in which trained practitioners are seldom available. Thus, this online GKT program has much potential for dissemination as a practical tool for the prevention of suicide in schools such as universities. Once we find the online GKT program sufficiently effective, it will contribute to the dissemination of gatekeeper online training to a large number of students to prevent suicide at a low cost and, as in the COVID-19 era, where close social contact is limited.

The main findings of this study will be disseminated via publications in peer-reviewed international journals. Study findings will also be presented at scientific conferences.

### Limitations

Due to the intervention’s nature, it is not possible for both the intervention implementer and participants to be blinded to group assignments. Because all the outcomes in this study will be obtained by the self-reported questionnaire, information bias could be introduced. Generalizability will be limited because snowball sampling will be used instead of random sampling.

### Conclusion

This is the first study to investigate the effectiveness of an online GKT program for students to improve self-efficacy as a gatekeeper using an RCT design. The study explored the potential of an online peer gatekeeper program for students that can be disseminated online to a large number of students with minimal cost.

## Appendix

Multimedia Appendix 1 ACT questionnaire.

## Abbreviations

CIUS: Compulsive Internet Use Scale
FCV-19S: Fear of COVID-19 Scale
G7: Group of 7
GKSES: Gatekeeper Self-Efficacy Scale
GKT: gatekeeper training
iOSDMH: Implementation Outcome Scales for Digital Mental Health
K6: Kessler Psychological Distress Scale
LOSS: Literacy of Suicide Scale
MIDUS: Mental Illness and Disorder Understanding Scale
PPI: patient and public involvement
RCT: randomized controlled trial
SOC: sense of coherence
SOC3-UTHS: University of Tokyo Health Sociology version of SOC3 scale
SPIRIT: Standard Protocol Items: Recommendations for Interventional Trials
TPB: theory of planned behavior
