# Supplementary material for: Effectiveness of an Online Peer Gatekeeper Training Program for Postsecondary Students on Suicide Prevention in Japan: Protocol for a Randomized Controlled Trial
Source: JMIR Res Protoc. 2022 Apr 26;11(4):e34832. doi: 10.2196/34832 (PMC9092229; doi:10.2196/34832)
Supplement: Multimedia Appendix 1 [file resprot_v11i4e34832_app1.docx]

Multimedia Appendix 1. ACT Questionnaire.

Please select the most appropriate response regarding the last 2 months.

1. How many times have you asked an acquaintance (friend) whether they were considering suicide?
   1. None
   2. 1 time
   3. 2 times
   4. 3 times
   5. 4 or more times
2. How many times have you asked an acquaintance (friend) about their distress or depressed mood?
   1. None
   2. 1 time
   3. 2 times
   4. 3 times
   5. 4 or more times
3. How often have you asked a worrisome acquaintance (friend) about suicidal ideation?
   1. Never
   2. Rarely
   3. Sometimes
   4. Almost always
   5. Always
4. How often have you spent a while listening to the story of an anxious acquaintance (friend)?
   1. Never
   2. Rarely
   3. Sometimes
   4. Almost always
   5. Always
5. How often have you taught your anxious acquaintance (friend) how to use the resource with an expert?
   1. Never
   2. Rarely
   3. Sometimes
   4. Almost always
   5. Always
6. How often have you taught your anxious acquaintance (friend) how to use resources other than specialists?
   1. Never
   2. Rarely
   3. Sometimes
   4. Almost always
   5. Always
7. How often have you told your anxious acquaintance (friend) to ask for help when they were in trouble?
   1. Never
   2. Rarely
   3. Sometimes
   4. Almost always
   5. Always
8. How often have you taken an anxious acquaintance (friend) to a resource with specialists?
   1. Never
   2. Rarely
   3. Sometimes
   4. Almost always
   5. Always
9. How often have you taken an anxious acquaintance (friend) to a resource by nonspecialists for consulting?
   1. Never
   2. Rarely
   3. Sometimes
   4. Almost always
   5. Always
10. How often have you realized an acquaintance’s (friend’s) possibility of suicide danger signs?
    1. Never
    2. Rarely
    3. Sometimes
    4. Almost always
    5. Always
11. How often have you reached out to an anxious acquaintance (friend) (including email and SMS exchanges)?
    1. Never
    2. Rarely
    3. Sometimes
    4. Almost always
    5. Always
